# Comparison of a ready-to-use intranasal dexmedetomidine spray with traditional intranasal dexmedetomidine drops for sedation in preschool children: a prospective, randomized, controlled study

**DOI:** 10.3389/fphar.2025.1528612

**Published:** 2025-01-23

**Authors:** Qi-Qi Jin, Wei-Cha Cai, Ying-Feng Zhou, Yan-Tong Zhang, Gang Chen, Meng-Ting Xu, Jun Li, Kai-Ming Yuan

**Affiliations:** Department of Anesthesiology and Perioperative Medicine, The Second Affiliated Hospital and Yuying Children’s Hospital of Wenzhou Medical University, Key Laboratory of Pediatric Anesthesiology, Ministry of Education, Wenzhou Medical University, Key Laboratory of Anesthesiology of Zhejiang Province, Wenzhou Medical University, Wenzhou, Zhejiang, China

**Keywords:** atomization, m-YPAS, pediatric, preoperative anxiety, Ramsay sedation scale

## Abstract

**Purpose:**

This study compared the efficacy and acceptability of a ready-to-use intranasal dexmedetomidine spray (DS) versus traditional drops administered by syringe (DD) in pediatric patients undergoing elective surgery.

**Patients and Methods:**

Eighty-six preschool children were enrolled in a prospective, randomized, controlled study. Children were randomly assigned to receive either DS or DD. For children weighing between 10.5 and 18.5 kg, a dexmedetomidine dosage of 30 μg (two sprays) was administered, while those weighing between 18.5 and 25.5 kg received 45 μg (three sprays). In the DD group, dexmedetomidine was administered at a dose of 2 μg/kg based on body weight. The primary outcome was the proportion of children achieving a Ramsay sedation scale (RSS) score of ≥3 within 30 min. Secondary outcomes included acceptance of intranasal medication, anxiety at parental separation and prior to induction, and compliance with induction.

**Results:**

A total of 83 cases were analyzed. The proportion of children achieving an RSS score of ≥3 within 30 min was similar between the DS and DD groups (90.7% vs. 77.5%, respectively). However, the acceptance score was significantly better in the DS group (mean difference [95%]: −0.9 [−1.267 to −0.5325], P < 0.001). No significant differences were observed between the groups in terms of successful child-parent separation (88.4% vs. 85%) or satisfactory anxiolytic effect prior to induction (95.3% vs. 92.5%). Compliance with induction was comparable, with 53.5% in the DS group and 40.0% in the DD group demonstrating “optimal” compliance.

**Conclusion:**

Both intranasal spray and syringe drop methods were highly effective in providing sedation and anxiolysis, but the ready-to-use intranasal dexmedetomidine spray was more acceptable to children, offering a viable alternative to the syringe method.

**Clinical Trial Registration:**

ChiCTR.org.cn, identifier ChiCTR2400089374.

## 1 Introduction

The incidence of pre-anesthesia anxiety in children is as high as 80%. Consequently, difficulties in parental separation, establishing intravenous access, and inducing anesthesia are common. To improve children’s cooperation and minimize adverse psychological and behavioral complications, such as emergence agitation (Kain et al., 2004) and negative postoperative behavioral changes (Batuman et al., 2016), preoperative anxiety must be effectively managed.

Dexmedetomidine, a highly selective α2-adrenoceptor agonist, is widely used as an anxiolytic drug. It produces a sedative effect by acting on the locus coeruleus in the central nervous system, inducing sedation akin to natural sleep (Ramaswamy et al., 2021), with fewer perioperative respiratory adverse events (Zhang et al., 2023). Due to these properties, dexmedetomidine is frequently employed for pediatric sedation across various settings (Jackson et al., 2022; Shen et al., 2022).

The intranasal administration of dexmedetomidine, either via an atomizer or drops from a syringe, is preferred due to its high bioavailability and ease of use. The intranasal route not only avoids first-pass metabolism but also ensures rapid onset of therapeutic effects owing to the rich vascular plexus of the nasal cavity, which communicates with the subarachnoid space via the olfactory and trigeminal nerves (Trevino et al., 2020). Although atomization theoretically allows for more even drug distribution across the nasal mucosa, the bioavailability of intranasal dexmedetomidine reported in different studies varies significantly, ranging from 40% to 84% (Iirola et al., 2011; Miller et al., 2018; Li et al., 2018), with no clear advantage of atomization over drops (Li et al., 2018). Furthermore, clinical studies have not consistently demonstrated the superiority of atomized administration (Li et al., 2016; Xie et al., 2017). Atomizers for intranasal dexmedetomidine described in the literature have yet to be introduced in mainland China, where intranasal drops administered by syringe are commonly used.

A recent development in pediatric care is a ready-to-use intranasal dexmedetomidine spray kit (Jiangsu Hengrui Medicine Co. Ltd., Jiangsu, China) (Gao et al., 2024), which delivers 15 μg of atomized dexmedetomidine per spray. This method may offer a simpler and more acceptable means of intranasal administration. However, it remains unclear whether fixed-dose sprays and weight-based dosage drops produce similar sedative effects. This study tested this hypothesis and evaluated the potential advantages of these two methods.

## 2 Materials and methods

### 2.1 Study design and patients

**FIGURE 1:**
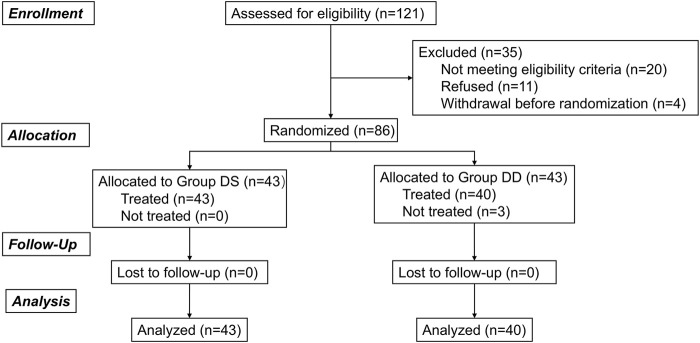
Flowchart of included and excluded patients.

This prospective, randomized controlled study was reviewed and approved by the Medical Ethics Committee of the Second Affiliated Hospital and Yuying Children’s Hospital of Wenzhou Medical University (Approval Number: 2023-K-218-02) and was registered at the Chinese Clinical Trial Registry (ChiCTR.org.cn; ChiCTR2400089374). Written informed consent was obtained from the children’s parents, and the children were encouraged to participate in the study. A total of 86 preschool children were enrolled between September 2024 and October 2024. The CONSORT flowchart is presented in Figure 1.

### 2.2 Inclusion and exclusion criteria

Children between 3 and 6 years of age, weighing 10.5–25.5 kg, and classified as American Society of Anesthesiologists (ASA) I or II, scheduled for elective tonsillectomy and adenoidectomy under general anesthesia, were eligible for inclusion.

Exclusion criteria included recent upper respiratory tract infection; cardiovascular disease; abnormal liver or kidney function; potential difficult airway; conditions rendering the child unsuitable for intranasal administration (e.g., excessive nasal secretions); and known allergies to dexmedetomidine.

### 2.3 Randomization and masking

Children were randomly assigned to receive either intranasal dexmedetomidine spray (Group DS) or drops (Group DD) from a syringe. Randomization was stratified by body weight in a 1:1 ratio: ≥10.5 kg and <18.5 kg, or ≥18.5 kg and ≤25.5 kg. An independent investigator generated the randomization list using a computer-based software. The randomization results were sealed in sequentially numbered opaque envelopes and kept by a trial coordinator. The envelopes were opened prior to intranasal administration as the order of enrollment.

Drug preparation and administration were performed by an independent assistant in a separate compartment within the preoperative preparation area. This assistant also evaluated the children’s acceptance of the medication and documented the results in a unique database, distinct from the general study database. The trial coordinator and assistant were not involved in the subsequent anesthesia management, perioperative care, or postoperative follow-up. The remaining evaluations were performed by two designated observers who were blinded to the drug administration. Prior to the study, these observers underwent standardized training in accordance with the standard operating procedures, ensuring consistency in both the execution and evaluation criteria for each assessment item. The healthcare team and the investigator responsible for patient recruitment, data collection, and follow-up assessments were blinded to group assignments. Unmasking was permitted only when clinically necessary.

### 2.4 Pre-anesthesia sedation and anesthesia

All children followed the hospital’s routine pre-anesthesia fasting protocol, which required fasting of clear liquids for 2 h, milk or light foods for 6 h, and solid foods for 8 h. Baseline characteristics, including age, sex, weight, height, and ASA status, were recorded prior to sedation. Preoperative anxiety was assessed using the Modified Yale Preoperative Anxiety Scale (m-YPAS).

Pediatric patients were sedated in the presence of their parents in the induction room. In Group DD, children were seated on their parents’ laps with their heads tilted backward, and intranasal dexmedetomidine was rapidly administered towards the top of the ear on the same side. In Group DS, the children’s heads were kept in a natural upright position as the spray kit needed to remain upright during use. Patients in Group DS received the ready-to-use dexmedetomidine nasal spray, with detailed spray kit parameters described by Gao et al. (2024). Dosage was stratified by body weight: children weighing between 10.5 kg and 18.5 kg received 30 μg (lower weight, two sprays), while those weighing between 18.5 kg and 25.5 kg received 45 μg (higher weight, three sprays). Group DD received intranasal dexmedetomidine drops using a needleless 1 mL syringe, prepared with a concentration of 100 μg/mL and dosed at 2 μg/kg according to body weight. A final volume of 0.2–0.3 mL per nostril was used as the ideal intranasal medication volume (Del Pizzo and Callahan, 2014). Children under 20 kg in Group DD received the drops in one nostril if the final volume was less than 0.4 mL; otherwise, the volume was equally divided between both nostrils.

Sedation levels were assessed using the Ramsay Sedation Scale (RSS), recorded before administration and at 10, 15, 20, 25, and 30 min after intranasal medication. Vital signs, including heart rate (HR) and blood oxygen saturation (SpO_2_), were monitored at 5-minute intervals. Bradycardia was defined as a heart rate reduction of more than 20% from baseline or below 70 beats per minute (Baum et al., 2016).

Thirty minutes after intranasal medication, the children were transferred to the operating room. For children who experienced difficulty with parental separation, additional methods were applied, including parental presence during transfer. Perioperative anesthesia management was performed according to institutional protocols. Briefly, preoxygenation was provided via mask, followed by incremental induction of anesthesia with sevoflurane to facilitate the establishment of venous access. General anesthesia was then induced intravenously using 2–3 mg/kg of propofol, 2 μg/kg of fentanyl, and 2 μg/kg of remifentanil. The children were intubated, and anesthesia was maintained with sevoflurane at a depth of 1–1.3 MAC. At the end of surgery, sevoflurane was discontinued. Postoperative analgesia was provided with an intravenous injection of pyroxene tromethamine (0.5 mg/kg), and 0.1 mg/kg of ondansetron was administered prophylactically to prevent postoperative nausea and vomiting. Extubation was performed once the standard criteria were met.

Following surgery, the children were transferred to the post-anesthesia care unit (PACU) for a minimum of 30 min of monitoring. Emergence agitation was diagnosed if the Pediatric Anesthesia Emergence Delirium Scale (PAED) score exceeded 10 (He et al., 2023), in which case 20 mg of propofol was administered intravenously. Children were discharged from the PACU when their modified Aldrete score was over 9.

### 2.5 Outcome assessments

The primary outcome of the study was the proportion of children achieving an RSS score of ≥3 within 30 min of intranasal administration. RSS was assessed every 5 min.

The secondary outcomes included the following:

1. Acceptance of intranasal medication, graded on a 4-point scale:
  −1: Crying after administration and unwilling to accept it again
  −2: Resistance and complaints of irritancy
  −3: Acceptable with minor discomfort
  −4: Well-accepted
2. Time to achieve RSS ≥3, observed at intervals of ≤20 min, 25 min, 30 min, or >30 min if RSS ≥3 was not achieved within 30 min.
3. Proportion of children achieving satisfactory child-parent separation after 30 min. Separation anxiety was assessed using the Parental Separation Anxiety Scale. A score of ≤2 points was labeled as satisfactory separation, indicating cooperation, unafraid behavior, or slight fear easily eased.
4. Proportion of cases achieving satisfactory anxiolytic effect prior to induction. Preoperative anxiety was evaluated with the m-YPAS both before intranasal medication and prior to anesthesia induction. The m-YPAS consists of 27 items across five categories: activity, emotional expressivity, state of apparent arousal, vocalizations, and use of parents. A satisfactory m-YPAS score was defined as less than 30 (Cai et al., 2024).
5. Anxiety and distress behaviors during anesthesia induction, assessed using the Induction Compliance Checklist (ICC) (Winterberg et al., 2020). The ICC is an observational scale with 10 negative behaviors during induction, scored 0–10. An ICC score of 0 was considered optimal compliance, while a score ≥4 indicated poor compliance.
6. Time of emergence from anesthesia, defined as the time from sevoflurane cessation to spontaneous eye opening.
7. Incidence of emergence agitation, evaluated using the PAED scale.
8. Length of PACU stay.
9. Adverse events, including bradycardia and respiratory complications.

### 2.6 Statistical analysis

The sample size was calculated based on findings from a pilot study and prior research (Gao et al., 2024), indicating that 92% of patients receiving dexmedetomidine nasal spray and 70% of patients receiving dexmedetomidine drops achieved RSS ≥3 within 30 min. To achieve 80% power with a 5% Type I error and a 10% dropout rate, a sample size of 86 children was required.

Statistical analyses were conducted using IBM SPSS 16.0 (IBM Corp., Armonk, NY, United States). Normality and homogeneity of variances were tested using the Shapiro-Wilk and Levene’s tests, respectively. Continuous variables with normal distribution were expressed as mean ± standard deviation (SD), nonparametric data were presented as median [range], and categorical data were reported as number (percentage). Normally distributed data were compared using Student’s t-test, nonparametric data were analyzed with the Mann-Whitney U test, and categorical data, including the incidence of emergence agitation, were analyzed using the χ^2^ test or Fisher’s exact test, as appropriate. Furthermore, a two-way ANOVA was conducted to evaluate the effects of the method of intranasal medication and stratified body weight on the acceptance of the medication.

## 3 Results

### 3.1 Baseline characteristics and perioperative data

Of the 121 children screened, 86 eligible patients were enrolled and evenly assigned to the DS and DD groups. Three children in the DD group refused intranasal medication, but all other children received the treatments and were included in the final analysis. Demographics and baseline characteristics were generally well-balanced between the two groups (Table 1).

**TABLE 1:** Demographics and baseline characteristics.

	Group DS (n = 43)	Group DD (n = 40)	*t/χ* ^*2*^	*P*-value
Age, year	4.5 ± 1.1	4.8 ± 0.9	−1.445	0.152
Sex, n (%)			0.624	0.429
female	22 (51.2)	17 (42.5)		
male	21 (48.8)	23 (57.5)		
Weight, kg	18.7 ± 4.4	19.2 ± 3.4	−0.462	0.645
Weight stratification, n (%)			0.111	0.739
≥ 10.5 kg and < 18.5 kg	22 (51.1)	19 (47.5)		
≥ 18.5 kg and ≤ 25.5 kg	21 (48.9)	21 (52.5)		
Operation duration	16.8 ± 6.8	15.0 ± 4.4	1.447	0.152
Anesthesia duration	33.3 ± 10.5	29.5 ± 8.6	1.798	0.076
RSS at baseline	1.7 ± 0.5	1.9 ± 0.5	−1.438	0.154
mYpas at baseline	36.8 ± 15.8	36.5 ± 15.7	0.109	0.913

Notes: Date are presented as mean (SD) or n (%).

Abbreviations: RSS, ramsay sedation scale; mYpas, the modified yale preoperative anxiety scale. SD, standard deviation.

### 3.2 Primary outcome

The proportion of children achieving an RSS score of ≥3 within 30 min did not differ significantly between the two groups, with 90.7% in the DS group and 77.5% in the DD group (Table 2).

**TABLE 2:** Primary and secondary outcomes.

	Group DS (n = 43)	Group DD (n = 40)	*t/χ* ^*2*^	*P-*value
Cases achieved RSS ≥ 3 within 30 min, n (%)	39 (90.7)	31 (77.5)	2.732	0.098
≥10.5 kg and <18.5 kg	18 (81.8)	13 (68.4)	0.992	0.319
≥18.5 kg and ≤25.5 kg	21 (100)	18 (85.7)	3.231	0.072
Acceptance of intranasal medication	3.8 ± 0.5	3.0 ± 1.1	4.315	<0.001
Cases achieved desired child-parent separation, n (%)	38 (88.4)	34 (85.0)	0.205	0.651
Minimal time for observed RSS ≧ 3, n (%)			3.162	0.367
≤20 min	15 (34.9)	13 (32.5)		
25 min	18 (41.9)	9 (30.0)		
30 min	6 (14.0)	9 (15.0)		
>30 min	4 (9.3)	9 (22.5)		
Cases achieved “Satisfactory” anxiolytic effect prior to induction, n (%)	41 (95.3)	37 (92.5)	0.297	0.586
Compliance of induction measured by ICC score, n (%)			3.425	0.180
Optimal	23 (53.5)	16 (40.0)		
Median	12 (27.9)	19 (47.5)		
Poor	8 (18.6)	5 (12.5)		
Time of emergence from anesthesia, min	44.3 ± 27.1	45.3 ± 18.2	−0.195	0.846
Incidence of emergence agitation, n (%)	4 (9.3)	3 (7.5)	NA^a^	1.000
Length of stay in PACU, min	49.4 ± 21.5	47.3 ± 18.3	0.482	0.631

Notes: Date are presented as mean (SD) or n (%).

^a^ *Fisher’s* exact test was applied.

Abbreviations: RSS, ramsay sedation scale; ICC, induction compliance checklist; PACU, post-anesthesia care unit; SD, standard deviation.

### 3.3 Secondary outcome

**FIGURE 2:**
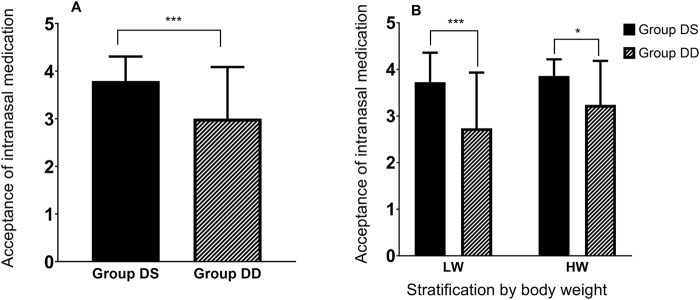
Acceptance of intranasal medication. **(A)** Comparison of intranasal medication acceptance between Group DS and Group DD. **(B)** The effect of intranasal medication method (DS or DD) and stratified body weight (LW or HW) on acceptance, analyzed using two-way ANOVA. LW (lower body weight), ≥10.5 kg and <18.5 kg; HW (higher body weight), ≥18.5 kg and ≤25.5 kg. Data are presented Mean ± SD. Asterisk denotes significant differences between groups at *P < 0.05 or ***P < 0.001.

Secondary efficacy outcomes are summarized in Table 2 and Figure 2. Nasal spray administration using the ready-to-use kit was more acceptable to children than the drops, as evidenced by a significantly lower acceptance score in the DD group compared to the DS group (mean difference [95% CI]: −0.9 [−1.267 to −0.5325], *P* < 0.001). A subsequent two-way ANOVA revealed that the acceptance of intranasal medication was statistically influenced by the method of administration (F [1, 79] = 19.45, P < 0.001), but not by body weight stratification (F [1, 79] = 2.990, P = 0.0877). No interaction effect was observed between the two factors (F [1, 79] = 1.036, P = 0.312), as shown in Figure 2. Additionally, no child reported irritation during the intranasal administration of dexmedetomidine or experienced symptoms of agitation afterward in either group.

The time to achieve an RSS score of ≥3 was comparable between the two groups. When stratified by body weight, the results remained comparable between the two groups across each body weight range. Most children achieved the desired child-parent separation, with 88.4% in the DS group and 85% in the DD group, though the difference was not statistically significant. Similarly, nearly all children demonstrated a satisfactory anxiolytic effect prior to anesthesia induction, with 95.3% in the DS group and 92.5% in the DD group, without any significant difference between the groups.

Compliance during induction, assessed using the ICC, also showed no significant difference. “optimal” compliance was observed in 53.5% of children in the DS group and 40.0% in the DD group, while “poor” compliance was recorded in 18.6% of the DS group and 12.5% of the DD group. No significant differences were found in the incidence of emergence agitation, time to emergence from anesthesia, or length of PACU stay.

### 3.4 Perioperative adverse events

No severe adverse events were observed. The frequency of adverse events was low and similar between two groups. Postoperative hypoxemia occurred in four cases in the PACU (2 cases in each group), all of which resolved after oxygen therapy. One child in the DS group experienced transient laryngospasm during extubation, which was successfully managed with oxygen therapy and assisted ventilation.

## 4 Discussion

Intranasal dexmedetomidine is commonly used for pediatric sedation (de Rover et al., 2023), and administration via needleless syringe is a widely adopted off-label method (Li et al., 2016; Xie et al., 2017; He et al., 2023). In this study, we compared the efficacy of dexmedetomidine spray from a ready-to-use kit with the more conventional syringe drop method. Both methods were highly effective, with no significant difference in the primary outcome, which was the proportion of children achieving an RSS ≥3 within 30 min. Similarly, the time to reach RSS ≥3 was comparable between the two groups.

However, children found the nasal spray more acceptable than the drops, as indicated by a significantly better acceptance score in the DS group. There were no significant differences in child-parent separation, anxiolytic effects, or induction compliance between the two methods. It appears that the ratios of cases achieved an RSS ≥3 within 30 min and cases achieved desired child-parent separation are discrepant. Although there is a strong correlation between RSS ≥3 and successful child-parent separation, we found that some children were able to comply with separation even RSS <3, while continued to have difficulty with separation at an RSS of 3.

The dosage of dexmedetomidine in this study was not intentionally consistent between the two methods, reflecting typical clinical practice. For the spray kit, each spray delivered 15 μg of dexmedetomidine in 50 μL, leading to a dosage range of 1.62–2.86 μg/kg (mean: 1.99 ± 0.23 μg/kg) for lower body weight children and 1.76–2.43 μg/kg (mean: 2.06 ± 0.29 μg/kg) for higher body weight children. The fixed dosage for the drops was 2 μg/kg. Despite this variation, the pre-anesthesia sedative effects of both methods were comparable, possibly due to dexmedetomidine’s wide therapeutic window, where small deviations in dosage may be negligible.

The wide therapeutic window of dexmedetomidine is well-supported by numerous clinical trials. Intranasal dexmedetomidine dosages for pediatric patients vary, typically ranging from 1 to 4 μg/mL, depending on the intended use (He et al., 2023). For example, a low dose of 1 μg/mL has been shown to effectively prevent emergence agitation (Kain et al., 2004), while a median dose of 2 μg/mL is commonly used for general sedation, i.e., for alleviating preoperative anxiety (Cai et al., 2024; Bromfalk et al., 2021), minimizing responses to venous cannulation (Xie et al., 2017), and facilitating sedation for transthoracic echocardiography (Panda et al., 2021). Higher doses are often required for magnetic resonance imaging (MRI) due to factors such as the high auditory stimulus, with doses ranging from 2 to 4 μg/mL in previous studies (de Rover et al., 2023).

Aside of pre-anesthesia sedation, dose dependent intranasal dexmedetomidine raise general concerns. Side effects are typically associated with high intranasal doses. The maximum dose in the present study was calculated to be 2.73 μg/kg. While intranasal dexmedetomidine may prolong the length of stay in the PACU, a recent meta-analysis revealed no statistical difference between doses of 1.5 μg/kg and 2 μg/kg (Hu et al., 2024). According to Tsze’s research, the sedation time for high doses of intranasal dexmedetomidine, ranging from 2 μg/kg to 4 μg/kg or higher, increased only slightly with each 1 μg/kg increment (Tsze et al., 2023). Taken together, these findings may explain the similar length of stay in the PACU observed in this study. Severe adverse effects, such as bradycardia and oversedation, are rare even at higher doses (Tsze et al., 2023); however, careful monitoring is recommended when higher doses are used. Beneficial effects are generally associated with appropriate dosing. It has been reported that the 95% effective dose of intranasal dexmedetomidine for preventing emergence agitation in children over 3 years old is 1.78 μg/kg (Lei et al., 2022). These additional effects may be further assessed in studies with larger sample sizes.

Notably, the ready-to-use intranasal dexmedetomidine spray was more acceptable to children in this study, as three children refused the drop administered by syringe. Fear and anxiety are prevalent among children receiving medical care, often stemming from interactions with healthcare providers, the clinical environment, and medical instruments. Reducing this emotional burden through distraction attention can improve patient compliance. Methods such as playing interactive games via virtual reality headsets or watching movies on TV or through virtual reality have been shown to be effective, as demonstrated by studies utilizing virtual reality (Jung et al., 2021) and/or digital media techniques (Hoge et al., 2017). Syringes, even needleless ones, may trigger associations with injection pain. In contrast, a spray pump resembles non-medical devices, avoiding negative associations with medical procedures. Moreover, the atomized sprays generated finer particles, likely reducing nasal irritation, potentially contributing to the higher acceptability of the spray method. The combination of virtual reality and intranasal sedation holds promise, given their complementary benefits.

Both nasal spray and syringe drop methods were highly effective in alleviating preoperative anxiety, as the majority of children in both groups achieved desired child-parent separation, with proportions exceeding 85%, and nearly all achieved a satisfactory anxiolytic effect prior to induction. The results suggested that both methods were equally effective of facilitating preoperative care and managing anxiety in pediatric patients. The slightly lower rate of child-parent separation in the spray group compared to a previous study by Gao et al. (2024). may be attributed to the shorter observation period (30 min vs. 45 min) (Gao et al., 2024). In this study, a 30-minute observation period was selected based on the typical onset time of dexmedetomidine, which is around 25 min (Yuen et al., 2012), making a duration of 30 min acceptable (Xie et al., 2017; He et al., 2023; Cai et al., 2024), as well as practical considerations regarding anesthesia efficiency and patient turnover.

Several limitations should be acknowledged. First, the physical differences between the spray kit and syringe made it impossible to blind the children or the independent assistant responsible for evaluating acceptance of the intranasal medication. This introduced a potential for performance and detection bias. However, other healthcare team members and the investigator responsible for patient recruitment, data collection, and follow-up assessments were blinded to group assignment. Second, the relatively small sample size may have affected the statistical power of some secondary outcomes, either overestimating or underestimating their significance. Third, although the syringe drop method with doses stratified by body weight is not a standard practice in clinical settings, an equivalence trial could be designed to isolate the effect of the delivery mode by comparing its efficacy with the other two methods. Fourth, due to the subsequent general anesthesia, sustained anxiolysis or delayed adverse effects could not be observed within the 30-minute period. Procedure sedation only, such as sedation for chest ultrasound, may serve as a suitable alternative to evaluate these effects. Lastly, this was a single-center study, and while efforts were made to standardize the procedure, including limiting the intranasal drop volume to 0.4 mL per nostril to optimize bioavailability, the results may not be generalizable. A multicenter trial that allows for variations in ordinary clinical practice is recommended to better compare these two methods.

## 5 Conclusion

Both the ready-to-use intranasal dexmedetomidine spray and the intranasal drop by syringe demonstrated high efficacy. However, the spray was more acceptable to children and produced comparable sedative and anxiolytic effects, making it a practical alternative to the syringe method.

## Data Availability

The raw data supporting the conclusions of this article will be made available by the authors, without undue reservation.
